# 2759. Cefiderocol vs Best Available Therapy for the Treatment of Carbapenem- Resistant *Acinetobacter baumannii*

**DOI:** 10.1093/ofid/ofad500.2370

**Published:** 2023-11-27

**Authors:** Gabrielle N Daisey, Nikunj M Vyas

**Affiliations:** Jefferson Health New Jersey, Blackwood, New Jersey; Jefferson Health New Jersey, Blackwood, New Jersey

## Abstract

**Background:**

Cefiderocol has been evaluated as a treatment of carbapenem-resistant gram negative infections. However robust data for the optimal approach to treat carbapenem-resistant *Acinetobacter baumannii* (CRAB) is lacking. The purpose of this study was to evaluate the efficacy of cefiderocol vs best available therapy (BAT) for the treatment CRAB infections.

**Methods:**

This IRB-approved retrospective observational review was conducted at a three-hospital community health system between June, 2020 to February, 2021. Hospitalized patients were included if they were ≥18 years old and received ≥24 hours of targeted antibiotics for CRAB. Patients were excluded if they were considered to be colonized or were pregnant. Patients in the cefiderocol group were matched with patients in BAT group based on age and source of infection in a 1:3 ratio. The primary endpoint of this study was to assess all-cause inpatient mortality (ACIM) at the end of therapy. The secondary endpoint included clinical cure (CC) at the end of therapy. A subgroup analysis was performed for patients treated with cefiderocol monotherapy, cefiderocol combination therapy, and combination BAT to determine differences in ACIM and CC.

**Results:**

A total of 60 patients were included in the primary analysis; 15 receiving cefiderocol and 45 receiving BAT. There was no difference in patient age, race, and diagnosis between both groups. Patients in the BAT group had a shorter length of stay vs cefiderocol group (14 vs 19 days. P=0.05). Ampicillin/sulbactam (86.7% vs 57.8%, P=0.06) and minocycline (86.7% vs 22.2%, P=0.0001) non-susceptible CRAB isolates were more common in the cefiderocol group vs BAT group (as seen in Figure 1). No differences were seen in ACIM (27% vs 18%, P=0.47) and CC (53% vs 60%, P=1.0) between both groups. The subgroup analysis revealed cefiderocol combination therapy had a decreased number of patients with ACIM and had favorable CC outcomes compared to cefiderocol monotherapy and combination BAT (as seen in Figure 2).

Comparison of Susceptibility Patterns

**Figure:**
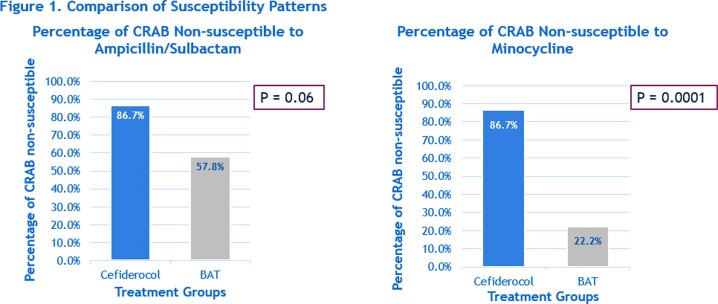

All Cause Inpatient Mortality and Clinical Cure in Subgroup Analysis of Monotherapy vs Combination Therapy

**Figure:**
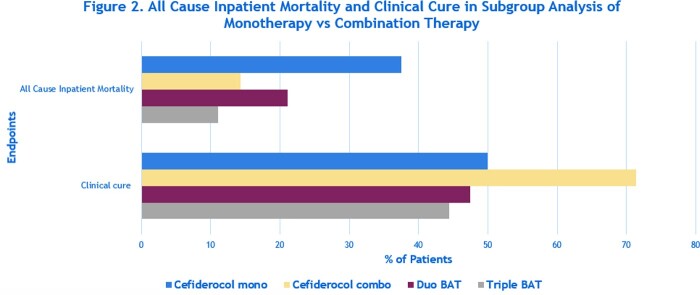

**Conclusion:**

Cefiderocol seems to be a viable option for treatment of CRAB infections. No differences were seen in ACIM and CC between cefiderocol and BAT. Cefiderocol combination therapy seems to be a preferred therapeutic option for CRAB infections.

**Disclosures:**

**All Authors**: No reported disclosures

